# The Effects of Chemotherapeutics on the Ovarian Cancer Microenvironment

**DOI:** 10.3390/cancers13133136

**Published:** 2021-06-23

**Authors:** Mark A. Eckert, Carlos Orozco, Jason Xiao, Melissa Javellana, Ernst Lengyel

**Affiliations:** Department of Obstetrics and Gynecology/Section of Gynecologic Oncology, University of Chicago, Chicago, IL 60637, USA; meckert@bsd.uchicago.edu (M.A.E.); seifer121@gmail.com (C.O.); Jason.Xiao@uchospitals.edu (J.X.); Melissa.Javellana@uchospitals.edu (M.J.)

**Keywords:** tumor microenvironment, stroma, cancer-associated fibroblasts, ovarian cancer, cancer therapy, carboplatin, taxanes, PARP inhibitors, review

## Abstract

**Simple Summary:**

Cancer cells are the target of most approved therapies. A growing body of evidence suggests that these agents have important roles in modulating the biology of host cells and their interactions with cancer cells, including blood vessels, fibroblasts, immune and fat cells, among others. This review provides an overview of potential roles of commonly used therapeutics in the tumor microenvironment, with a focus on cancer-associated fibroblasts. This includes an emphasis on therapies commonly used for the treatment of high-grade serous ovarian cancers (e.g., platinum, taxanes, PARP inhibitors, and anti-angiogenic agents). In vitro, in vivo, and clinical studies are included, and perspectives offered on how to best interpret the influence of therapeutics on normal cells.

**Abstract:**

High-grade serous ovarian cancer (HGSOC) is characterized by a complex and dynamic tumor microenvironment (TME) composed of cancer-associated fibroblasts (CAFs), immune cells, endothelial cells, and adipocytes. Although most approved therapies target cancer cells, a growing body of evidence suggests that chemotherapeutic agents have an important role in regulating the biology of the diverse cells that compose the TME. Understanding how non-transformed cells respond and adapt to established therapeutics is necessary to completely comprehend their action and develop novel therapeutics that interrupt undesired tumor–stroma interactions. Here, we review the effects of chemotherapeutic agents on normal cellular components of the host-derived TME focusing on CAFs. We concentrate on therapies used in the treatment of HGSOC and synthesize findings from studies focusing on other cancer types and benign tissues. Agents such as platinum derivatives, taxanes, and PARP inhibitors broadly affect the TME and promote or inhibit the pro-tumorigenic roles of CAFs by modifying the bidirectional cross-talk between tumor and stromal cells in the tumor organ. While most chemotherapy research focuses on cancer cells, these studies emphasize the need to consider all cell types within the tumor organ when evaluating chemotherapeutics.

## 1. Introduction

High-grade serous ovarian cancer (HGSOC) is the most lethal gynecologic malignancy. It is diagnosed at an advanced stage in 75% of women, which substantially contributes to the poor five-year survival of less than 50%. Despite aggressive treatment with cytoreductive surgery and platinum/taxane-based chemotherapy, 70–85% of patients with HGSOC will experience a recurrence [1]. Furthermore, the chemotherapeutic agents used during treatment can elicit serious off-target effects, including fatigue, myelosuppression, and neuropathy [2]. A small but growing body of literature has identified important biological effects of chemotherapeutics on the non-transformed cells of the tumor microenvironment (TME) [3]. Several chemotherapeutic agents can regulate cancer-associated fibroblasts (CAFs) by modifying their biology in ways that either enhance or restrain their pro-tumorigenic behavior. Dissecting these changes will help us better understand how chemotherapy influences non-malignant host cell populations to modulate disease progression, therapeutic response, and side effects of therapy.

**Figure 1:**
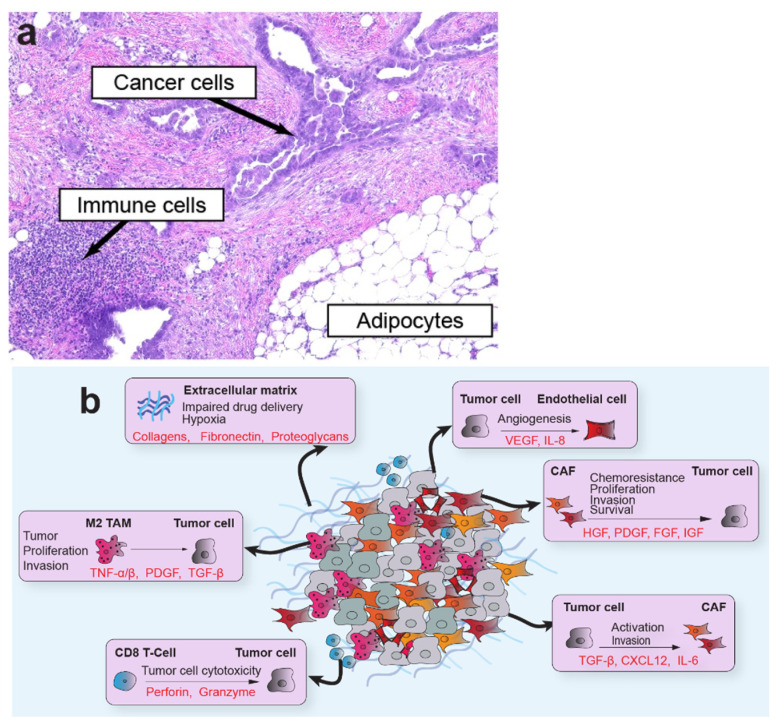
Cellular heterogeneity and bidirectional communication in the TME. (**a**) Representative image of a high-grade serous ovarian cancer metastasis to the omentum exemplifies the diverse ecosystem of tumors that includes cancer cells, immune cells, CAFs, adipocytes, endothelial cells, and other cells embedded in an ECM. CAFs and endothelial cells are present throughout the TME. (1:100) (**b**) Cross-talk between cell types in the ECM involves bidirectional signaling between tumor and stromal cell types that enforce a pro-tumorigenic microenvironment.

The TME is a multicellular network of tumor cells and host-derived cells, including CAFs, fibroblasts, myofibroblasts, adipocytes, endothelial cells, and immune cells, embedded in a distinct extracellular matrix (ECM; Figure 1). The host cells often comprise the most significant proportion of cells within the tumor organ. As one of the most abundant cellular components of the TME, CAFs are of particular interest in tumor progression [4]. CAFs encompass a highly dynamic populations of activated fibroblasts with pleiotropic functions that shape tumor behavior and engage in a complex network of cross-talk with cellular and non-cellular components of the TME. Their rich secretome supports interactions with multiple cell types in the TME to induce tumor growth, epithelial-to-mesenchymal transition (EMT), angiogenesis, immunosuppression, and ECM remodeling. These elongated cells do not express epithelial, endothelial, or leukocyte markers, and generally maintain stable genomes that lack mutations found in nearby tumor cells [5]. In addition, reactive oxygen species secreted by tumor cells alter CAF metabolism and induce oxidative stress, autophagy, and upregulation of glycolytic enzymes [6]. Although most studies support a pro-tumorigenic role for CAFs in the TME, other studies have found compelling evidence that, in some situations or model systems, CAFs restrain tumor progression [7,8,9]. Several markers have been used to characterize distinct subtypes of CAFs, with smooth muscle actin (αSMA) and fibroblastic activation protein (FAP) among the most widely used. Other markers, such as platelet-derived growth factor receptor beta (PDGFR-β), vimentin, caveolin 1, CD10, GPR77, and tenascin C, are also useful to resolve organ-specific CAF subtypes. Several comprehensive reviews addressing CAF origins, functions, and subtypes can be found elsewhere [4,5,8].

Advanced HGSOC is treated with an aggressive combination of chemotherapy and surgery. Depending on tumor volume at presentation, patients with localized or low-volume metastatic disease undergo surgery followed by six cycles of chemotherapy. Patients with high-volume metastatic disease first receive three cycles of carboplatin and paclitaxel prior to surgery, with three additional cycles after surgery (“neoadjuvant chemotherapy”). A debulking surgery that leaves behind no visible disease confers the most survival benefit to the patient, and the choice of treatment sequence is weighted to maximize the benefit and minimize the morbidity of surgery [10,11]. Unfortunately, recurrence rates are high, being greater than 70%, and long-term survival is infrequent, with only 15% of women with advanced stage cancer surviving 7–10 years [12]. An important determinant of overall survival in HGSOC is persistent susceptibility of the disease to platinum-based agents [13]. Recurrence therapy is chosen based on the interval from the last platinum agent, overall health, acquired toxicities, germline/somatic mutations, and other factors. Second-line or maintenance treatments can involve anti-angiogenics, anti-metabolites, PARP (poly ADP-ribose polymerase) inhibitors, topoisomerase inhibitors, and occasionally radiation therapy [14,15,16,17,18]. The diverse treatment options used in patient care and clinical trials highlight the relevance of understanding their potential impact on the non-transformed components of the TME.

In this review, we assess experimental data that examines how commonly used ovarian cancer chemotherapeutics, including platinum derivatives, taxanes, PARP inhibitors, and anti-angiogenics, alter the function of genetically normal cells in the TME. Although we focus on therapies used in the treatment of ovarian cancer, we incorporate evidence from other histological types of cancer to broadly understand therapy-induced changes in CAF biology and their impact on modifying cancer progression across different tumor types. The challenges in understanding these complex interactions highlight the need for improved model systems that recapitulate the heterogeneity of the TME. Single-cell or compartment-resolved approaches, combined with high-fidelity preclinical models of cancer treatment, will be essential to unravel the biological and clinical relevance of these effects.

## 2. Alkylating Agents (Cisplatin and Carboplatin)

Alkylating agents, including cisplatin and carboplatin, act by forming DNA adducts and DNA strand cross links which lead to DNA breakage or cross-linking [19]. If a cell cannot repair the lesion, RNA synthesis and DNA replication stall, and the cell undergoes apoptosis. The most common and most efficient primary therapeutic regimen for HGSOC is carboplatin in combination with paclitaxel every three weeks for a total of six cycles [20]. Cisplatin can be used with equivalent efficacy, particularly in patients with myelosuppression that cannot be overcome with carboplatin dose reduction but is associated with a worse overall side effect profile [21]. Platinum compounds cross the fibroblast cell membrane via the CTR1 and CTR2 copper membrane transporters [22,23]. CAFs, in particular, express less CTR1 than adjacent normal fibroblasts and cancer cells, which contributes to their generally chemoresistant phenotype [24].

Platinum agents have been found to alter the CAF secretome, inducing the secretion of protease inhibitors, cytokines, and miRNA-containing exosomes. In primary esophageal squamous cell cancer (ESCC), cisplatin-treated CAFs secreted high levels of plasminogen activator inhibitor-1 (PAI-1), which subsequently promoted cancer cell proliferation and protection from cisplatin-induced apoptosis via inhibition of caspase-3 and activation of AKT and ERK1/2 pathways [25]. Clinical analysis of 49 ESCC patients indicated that those with high expression of PAI-1 in CAFs had significantly worse progression-free survival (PFS). Masuda et al. further found that in vitro inhibition of PAI-1 in lung CAFs increased cancer cell apoptosis and reduced CAF α-SMA expression. Treatment of a co-culture system with PAI-1 inhibitors consequently increased the efficacy of cisplatin killing cancer cells [26].

Multiple studies have found that platinum-based agents can regulate the cytokine profile of CAFs. Lung CAFs treated with cisplatin upregulated IL-11 expression in a time- and dose-dependent manner, promoting pro-survival STAT3 signaling in lung cancer cells [27,28]. In a longitudinal study of ovarian cancer patients from whom tumor tissue was collected before and after chemotherapy, levels of IL-6 were elevated in αSMA^+^ stromal cells following platinum treatment [29]. In vitro treatment of CAFs from primary human ovarian cancer with cisplatin enhanced their chemoprotective properties in an IL-6-dependent manner. Systematic profiling of cytokines upregulated by HGSOC CAFs following cisplatin treatment found significant elevation of CCL5, IL-8, and MIP [30]. Ovarian cancer cells treated with CCL5 showed decreased apoptosis when exposed to cisplatin, which was consistent with the finding that platinum-resistant patient samples expressed elevated CCL5 levels compared to platinum-sensitive samples. Moreover, CAFs may respond differently than normal fibroblasts to platinum agents, as was observed when oxaliplatin treatment upregulated sDTK, IL-17A, and TGF-β in CAFs but not in normal fibroblasts [31]. In colorectal cancer patient samples, IL-17A level was also found to increase following chemotherapy.

In addition to growth factors and cytokines, CAFs also secrete exosomes containing RNA molecules that may influence the gene expression profile and behavior of cancer and normal cells in the TME [32]. Cisplatin- or paclitaxel-treated gastric CAFs secreted exosomes containing high levels of miR-522, which suppressed ferroptosis in cancer cells and promoted chemo-resistance [33]. Although miR-522 expression in primary CAFs was not elevated, treated CAF exosomes contained nearly a four-fold higher level of miR-522, indicating a preferential enrichment of miR-522 in exosomes. Another miRNA, miR-196a, was upregulated in exosomes from cisplatin-treated head-and-neck cancer (HNC) CAFs, which ultimately enhanced the proliferation and chemo-resistance of HNC cells via targeting CDKN1B and ING5 [34].

Platinum-based interventions may also contribute to transforming normal fibroblasts into CAFs through increasing the expression of CAF-related markers such as FAP and α-SMA, altering the metabolic activity of normal fibroblasts, and inducing some aspects of senescence. Co-culture experiments with bladder cancer suggest that cisplatin accelerates normal fibroblasts’ transition to CAFs and increased the expression of α-SMA and FAP [24]. In immortalized human foreskin fibroblasts, cisplatin and carboplatin treatment increased L-lactate production and glucose consumption [35]. Using glycolytic flux analysis, the authors found that platinum treatment elevated fibroblast glycolysis and reduced oxygen consumption rates, indicating a metabolic switch. A secreted fibroblast factor, the ECM, and metabolism are linked in a report describing a novel role for the ECM protein collagen (COL) 11A1.The CAFs secrete COL11A1 that binds to the cancer cells through a discoidin receptor, which leads to upregulation of fatty acid oxidation, enabling the cancer cells to withstand carboplatin treatment [36].

Because some features of the CAF phenotype are similar to the senescence-associated secretory phenotype (SASP), it may be significant that platinum treatment has also been found to induce senescence and autophagy in CAFs. Treatment of human lung fibroblasts with platinum agents caused a prematurely senescent phenotype, evidenced by elevated p53 expression, loss of the membrane gap junction protein connexin 43 (Cx43), and morphological changes including flattening, filopodia extensions, and cytoplasmic vacuole formation [37]. Similarly, cisplatin treatment of normal oral fibroblasts and foreskin fibroblasts upregulated senescence markers and increased α-SMA expression [38]. An important consideration when interpreting these studies is that carboplatin and cisplatin may exert distinct effects on CAFs due to differences in uptake, bioactivity, and mechanism [39,40,41]. For example, carboplatin, but not cisplatin, was found to augment the glycolytic reserve, upregulate senescence and CAF markers, and promote HIF, SMAD, and STAT signaling in immortalized fibroblasts [35]. 

As CAFs comprise a highly heterogeneous cell population [42], CAF subpopulations may respond differently to platinum-based therapies. Indeed, Su et al. demonstrated that CD10^+^GPR77^+^ CAFs associated with poor prognosis also exhibited docetaxel and cisplatin resistance compared to CD10^−^GPR77^−^ CAFs [43]. When challenged with cisplatin, CD10^+^GPR77^+^ breast cancer CAFs demonstrated significantly lower levels of apoptosis and growth inhibition. Furthermore, not all studies have found that platinum agents have tumor-promoting effects on the TME. In a study focused on lung cancer, cisplatin attenuated the ability of CAFs to promote the adhesion and invasion of cancer cells and reduced cancer cell AKT and NF-κB signaling via an unidentified paracrine mechanism [44]. In vivo experiments demonstrated that while co-implantation of CAFs and lung cancer cells increased tumor volume by over seven-fold, co-implantation with cisplatin-treated CAFs was comparable to implantation of cancer cells alone. Mesothelial cell-derived CAFs in the omental TME decreased cisplatinum sensitivity of HGSOC cells through secretion of fibronectin, which induced the PI3K pathway in the cancer cells [45].

Fully elucidating the mechanistic effects of therapeutic agents on CAFs will require understanding CAF heterogeneity and how therapies might balance cell subpopulations. It is important to note that all these studies indicate that some chemotherapy effects are tumor-promoting.

The effects of alkylating agents on other cellular components of the TME also deserve further attention. Notably, many studies demonstrate alkylating agents cause bone marrow toxicity, myelosuppression, and inhibition of the self-renewal capacity of hematopoietic stem cells [46,47,48]. Cisplatin has multifactorial roles in promoting a tumor-suppressive immune response, including increasing the range of antigen recognition, enhancing macrophage tumoricidal ability, promoting Th1 cytokine secretion, and regulating the recruitment of M1 macrophages, T regulatory cells, and CD8+ T cells [49,50,51,52]. In adipocytes, cisplatin may increase lipolysis while impeding lipogenesis [53]. Platinum treatment of endothelial cells induces features associated with angiogenesis, including dose-dependent decreases in migration and upregulation of ICAM-1, VEGF, and several cytokines such as IL-1 and IL-6 [54,55,56,57]. Independently, CAFs can promote the leakiness of blood vessels through the secretion of microfibrillar-associated protein (MFAP) 5 which binds to integrin receptors present on endothelial cells [58]. All of these effects on other cell types in the TME may also have the potential to regulate the behavior of CAFs.

## 3. Paclitaxel and Docetaxel

Paclitaxel, a plant alkaloid derived initially from the yew tree, is a cornerstone of upfront and recurrent treatment of HGSOC. Taxanes act during the M phase of the cell cycle by binding to intracellular microtubules to promote their assembly and stabilization, thus disrupting mitosis and leading to cell death [19]. Docetaxel is a therapeutically equivalent choice with a lower risk of peripheral neuropathy [59]. Taxanes can be used in the recurrent setting in both platinum-sensitive and -resistant patients [60].

There is emerging evidence that, in many situations, CAFs cooperate with tumor cells to enhance resistance to anticancer treatments [61]. In response to chemotherapy, CAFs may secrete cytokines, metabolites, and exosomes to potentiate stemness, metabolic reprogramming, and pro-survival signaling in tumor cells to orchestrate chemo-resistance [62]. Co-culture of taxane-treated normal lung fibroblasts with non-small-cell lung carcinoma tumor cells led to increased paclitaxel resistance of cancer cells, suggesting that paclitaxel may promote chemo-resistance via paracrine signaling [63]. Taxane-based chemotherapeutics may also regulate other aspects of the CAF phenotype by transcriptionally attenuating pro-tumorigenic paracrine signaling. Treatment of primary human breast cancer CAFs with docetaxel led to significant decreases in the expression of CXCL2, MMP1, IL-8, FF1, and CXCR7, among other cytokines. Co-culture experiments revealed that docetaxel-treated CAFs promoted the adhesion, invasion, and proliferation of MDA-MB-231 breast cancer cells [64]. A study of primary breast CAFs treated with docetaxel found that upregulation of MMP-1 and collagen IV was important for mediating chemo-resistance through ECM remodeling [65]. Caution should be applied to the interpretation of any in vitro or ex vivo experiments, however, as one study found divergent effects of paclitaxel treatment on primary CAFs compared to intact tissue sections, with increased apoptosis of CAFs observed in the ex vivo model system [66].

Several studies have suggested that taxanes may also regulate the behavior of normal fibroblasts and promote some aspects of the CAF phenotype. In an approach using label-free, quantitative proteomics, treatment of benign foreskin fibroblasts led to profound changes in energy metabolism, autophagy, senescence, myofibroblastic differentiation, and expression of inflammatory markers [67]. This included increased expression of common CAF markers such as α-SMA, fibronectin, and vimentin and upregulation of interleukin 6 (IL-6) and STAT3 signaling. In parallel to the metabolic reprogramming that occurs in CAFs [4,68,69], taxane treatment of dermal fibroblasts increased glycolysis, autophagy, and pro-inflammatory signaling [35,67].

In contrast, studies investigating the effects of taxanes on normal fibroblasts in non-cancer conditions have found evidence of anti-fibrotic effects. In a model of extrahepatic bile duct fibrosis, treatment of human gallbladder myofibroblasts with paclitaxel led to decreased autocrine TGFβ-1 signaling and reduced collagen 1 production associated with fibrosis [70]. In another study examining renal interstitial fibrosis, paclitaxel treatment led to decreased SMAD signaling and suppression of pro-inflammatory cytokine production accompanied by a strong reduction in α-SMA and collagen 1 expression [71]. The mechanistic basis for the differential effects of taxanes on normal fibroblasts and CAFs has not yet been explored.

In addition to effects on fibroblast components of the TME, taxanes may also regulate endothelial cells by exerting intrinsic anti-angiogenic effects. Interestingly, human endothelial cells accumulate higher intracellular levels of paclitaxel than non-endothelial cells, suggesting selectivity and increased susceptibility of endothelial cells to the drug [72]. Multiple studies have found that taxanes can directly compromise endothelial cells by inducing apoptotic cell death [73,74,75,76]. Functionally, paclitaxel attenuates endothelial cell migration, inhibits endothelial tube formation [77], and induces some aspects of the senescent phenotype [78]. Although taxanes may reduce the angiogenic activities of endothelial cells, there is also evidence indicating paclitaxel can increase vascular endothelial growth factor (VEGF) production in cervical cancer tumor cells by regulating hypoxia-inducible factor 1α (HIF-1α) and NF-κB signaling, thereby increasing angiogenesis and promoting chemo-resistance [79].

## 4. Poly ADP-Ribose Polymerases Inhibitors

Poly ADP-ribose polymerases (PARPs) are enzymes critical for the repair of single-stranded DNA breaks, as well as contributing to the repair of double-stranded breaks and the stabilization of replications forks [80]. In cells with double-stranded DNA repair deficiencies, such as those with *BRCA1/2* gene mutations, PARP inhibition leads to synthetic lethality. PARP inhibitors (PARPi) are oral medications that act to trap or inhibit PARP enzymatic action. Maintenance PARPi treatment after upfront chemotherapy has recently proven effective at lowering the risk of disease progression or death by 70% at 41 months in HGSOC patients with a *BRCA1/2* mutation. They are now standard in the frontline setting [11,15].

Most studies examining the effects of PARP inhibition on stromal cell populations have focused on normal fibroblasts and found that PARPi affected the regulation of TNFα and TGF-β signaling. Whereas PARP inhibition attenuates the TNFα-induced fibroblast response, its impact on TGF-β mediated effects is less clear. Isolated fibroblast-like synoviocytes treated with TNFα and a PARPi (DPQ or ANI) had reduced expression of inflammatory mediators such as MMP-3, IL8, and MCP-1 [81]. This was associated with diminished TNFα-induced proliferation, JNK phosphorylation, and AP-1 and NF-kB binding. Similar trends were observed in another study which found that pretreating murine fibroblasts with the PARPi INH2BP also suppressed JNK activation and AP-1 DNA binding [82]. In murine fibroblasts, the PARPi 3AB reduced levels of TNF-induced ATP depletion and death [83]. Broadly, TNFα-induced signaling in fibroblasts is attenuated by PARPi.

In human skin fibroblasts, PARP inhibition by 3-AB increased the stimulatory effects of TGF-β, including upregulation of α-SMA transcription, expression, and stress fiber formation compared to TGF-β alone [84]. Additional synergistic effects included upregulation of collagen expression, increased collagen release, and elevated SMAD3 signaling. In vitro, treatment with 3-AB exacerbated fibrosis induced by topoisomerase or bleomycin, resulting in increased dermal thickness and hydroxyproline content. Other studies, however, indicate anti-fibrotic roles for PARP inhibition. Knockdown of PARP1 in cardiac fibroblasts repressed TGF-β1-induced proliferation, migration, and differentiation [85]. In rat models of myocardial infarction (MI), 4-AB alleviated fibrosis and reduced collagen deposition, with an associated decrease in α-SMA expression. In addition, 4-AB increased p62 levels and reduced the LC3-II/LC3-I ratio, suggesting that PARP inhibition may increase autophagy. These divergent phenotypes may be due to differences in cellular context, fibroblast type, or experimental design considerations.

PARPi also demonstrates profound immunomodulatory effects, promoting anti-tumor immune responses by upregulating cytotoxic immune cells such as CD8^+^ T-cells, B-cells, and NK cells, while decreasing the number of myeloid-derived suppressor cells [86,87]. This anti-tumor response is at least in part due to upregulation of the STING pathway by PARPi [88]. While PARPi show promise in cancer treatment, they are also associated, albeit rarely, with severe side effects such as myeloid leukemia and myelodysplastic syndrome [89]. Importantly, maintenance PARPi treatment can be of extended duration, for two or more years, raising the possibility that long-term, global inhibition of PARP may have distinct influences on the biology of normal cells.

## 5. Anti-Angiogenic Agents (Bevacizumab)

Bevacizumab, an antibody against VEGF and angiogenesis, is often used to treat recurrent HGSOC as an additional treatment option to target the angiogenic potential of the TME [90]. In upfront therapy for HGSOC, there is some evidence that it benefits poor-prognosis patients with high tumor volume, stage IV, or incompletely resected disease [91]. Bevacizumab is also used in combination with PARPi for maintenance therapy in the upfront setting, with a 19.5-month progression-free survival benefit [92]. A PFS benefit has been seen when used in combination therapy for both platinum-sensitive and -resistant recurrence [93,94]. The lack of a consistent overall survival benefit and the rare risk of serious adverse events, including gastrointestinal perforation, bleeding diathesis, and poor wound healing, has limited this agent’s use. Although anti-VEGF inhibitors are meant to target endothelial cells, fibroblasts also express the receptors VEGFR1 and VEGFR3 [95]. No studies have directly investigated the effects of anti-angiogenic agents on CAFs, but bevacizumab exerts some direct effects on normal fibroblast populations. Bevacizumab-treated rat conjunctival fibroblasts exhibit reduced growth, ECM remodeling, and metabolic activity associated with decreased expression of VEGF, VEGFR1, VEGFR2, TGF-β1, and TGF-β2 [96]. Pharmacologically blocking VEGFR in human lung fibroblasts from patients with idiopathic pulmonary fibrosis suppressed the proliferative effects of secreted factors, including PDGF and bFGF [97]. VEGFR inhibition upregulated pro-MMP-2 activity, downregulated TIMP-2 secretion, and suppressed TGF-β-induced collagen secretion. Another study examining human tendon fibroblasts found that treatment with bevacizumab reduced metabolic activity and viability in a dose-dependent manner [98]. Bevacizumab also decreased the expression of MMP-1, MMP-2, and laminin, suggesting that it may play a role in ECM remodeling.

## 6. Topoisomerase Inhibitors (Doxorubicin, Ropotecan, and Mitoxantrone)

Topoisomerase inhibitors, including doxorubicin, topotecan, and mitoxantrone, act by inhibiting topoisomerase enzymes, which are responsible for the winding of DNA, leading to DNA strand breaks [99]. Doxorubicin has the additional effect of distorting the DNA double helix and generating free radicals. Liposomal doxorubicin and topotecan are both used for recurrent ovarian cancer with limited efficacy [100]. Although the effects of topoisomerase inhibitors on CAFs have not been extensively investigated, several studies of normal fibroblasts suggest that topoisomerase inhibitors may regulate the TME. Fibroblasts appear to be sensitive to the topoisomerase II inhibitor mitoxantrone, with the treatment of dermal fibroblasts leading to senescence and enhanced glycolysis [101]. Mitoxantrone-treated fibroblasts have been found to induce a cancer stem cell phenotype in MCF7 cells, as assessed using a luciferase reporter system in tumor–stromal co-culture conditions [35]. Mitoxantrone treatment of prostate, ovarian, and breast primary CAFs upregulated WNT16B, thereby mediating acquired resistance, suggesting a role for mitoxantrone in the regulation of Wnt signaling [102]. In a study that directly examined the effects of topoisomerase inhibitors on CAFs in a mouse model of desmoplastic melanoma, a combination of mitoxantrone and the triterpenoid celastrol decreased CAF-mediated collagen production and was associated with a CD8 T-lymphocyte- and dendritic cell-mediated immunogenic response [103]. The effects of topoisomerase inhibitors on other components of the TME have not been systematically investigated.

## 7. Antimetabolites (Gemcitabine)

Gemcitabine is a pyrimidine analog that primarily acts by inhibiting DNA synthesis through direct incorporation into the DNA backbone. It may also induce activation of mitogen-activated protein kinase (MAPK), triggering apoptosis in response to cellular stress in tumor cells [104]. Gemcitabine is commonly used in the recurrent setting and has a single-agent response rate of about 19%. Common dose-limiting toxicities include neutropenia, nausea, appetite suppression, and a flu-like syndrome [105]. Relatively few studies have examined the effects of gemcitabine on stromal cell populations. CAFs appear to be resistant to gemcitabine treatment when compared to normal fibroblasts or cancer cells [106]. It has been suggested that scavenger molecules from pancreatic CAFs may modify gemcitabine accumulation in tumors by entrapping the active drug and reducing its delivery to cancer cells [107]. In co-culture model systems, pre-treatment of immortalized rat CAFs protected tumor cells from the cytotoxic effects of gemcitabine. Interestingly, inhibition of autophagy in CAFs with chloroquine also reduced cancer cell death in response to gemcitabine [108]. Several studies have also found roles for gemcitabine in the regulation of CAF exosome production. The treatment of pancreatic CAFs with gemcitabine resulted in the increased release of exosomes containing the EMT regulator Snail. Uptake of these exosomes by tumor cells reduced the cytotoxic effects of gemcitabine treatment [109]. Similarly, Fang et al. found that CAF-derived exosomes transferred miRNA-106b to tumor cells and regulated gemcitabine resistance in a TP53INP1-dependent manner [106].

## 8. Radiotherapy

Radiotherapy involves targeted high-frequency ionizing radiation which ejects electrons from atoms to create ions which then form free radicals, leading to DNA damage. The advent of highly active chemotherapy for HGSOC in the 1990s sidelined radiation therapy in this disease. Its current use is limited to treating isolated recurrences or for symptom palliation [18]. Radiation induces significant changes in the CAF secretome, including upregulation of factors such as IGF-1, bFGF, IL-6, IL-8, GRO, HDGF, and potentially HGF [110,111,112,113]. Furthermore, radiation promotes MMP-3 and possibly MMP-1 expression in CAFs [114,115]. Consistent with the role of CAFs in the regulation of ECM remodeling, it has also been reported that radiotherapy elevates CAF expression of integrins β1, α5, and, most significantly, α2 [114]. Irradiated CAFs produced a stiffer collagen matrix when grown in a 3D culture system [116]. Radiotherapy also increases premature senescence in CAFs [114,117]. Cumulatively, these data suggest that CAFs resist radiation treatment through acquiring a senescent phenotype and that they can simultaneously contribute to cancer cell resistance by secreting pro-tumorigenic factors and remodeling the ECM.

## 9. Discussion

**Figure 2:**
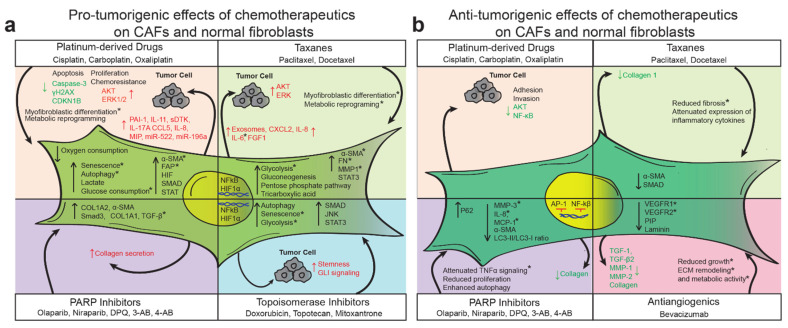
Effects of therapeutic agents on cancer associated fibroblasts (CAF)s. The diverse therapeutics used in the treatment of ovarian cancer, including platinum agents, taxanes, Poly ADP-ribose polymerases (PARP) inhibitors, tomoisomerase inhibitors, and anti-angiogenics, may have (**a**) pro-tumorigenic or (**b**) anti-tumorigenic effects on CAFs. * Experimental or clinical observations in normal fibroblasts or fibroblasts from disease states other than cancer.

Studies investigating the effects of anticancer agents on normal cells in the tumor microenvironment have focused on a wide range of molecular and phenotypic features, including metabolic and transcriptional reprogramming and intracellular signaling (Figure 2). These studies have primarily found that CAFs, in response to first-line chemotherapies, secrete multiple cytokines, metabolites, ECM-remodeling enzymes, and exosomes that transform the TME and generally promote chemo-resistance. Platinum derivatives and taxanes appear to promote a precancerous metabolic phenotype in stromal cells, resulting in augmented glycolysis, glucose consumption, lactate production, and activity of several pro-tumorigenic pathways [35,67]. In response to these agents, normal fibroblasts adopt at least some aspects of the CAF phenotype, but it is unclear if these changes are long-lasting or reversible [24,35,67,83]. Other therapies, including PARP inhibitors, anti-angiogenic agents, and topoisomerase inhibitors, have been less studied; nevertheless, some evidence suggests that they have TME-modifying capabilities. Of note, alongside cytotoxic chemotherapeutics, many patients receive 5-HT3 antagonists, steroids, and NK1 antagonists to manage nausea and emesis [118]. The effects of these agents on the TME have not been well investigated, although several stromal cell types express the relevant cognate receptors [119,120,121,122,123].

Additional research using more physiologically relevant models will be necessary if we are to illuminate the complex processes that occur in the treated TME. Organotypic model systems that incorporate both tumor and stromal cell types have proven to be particularly valuable in studies that reveal heterotypic cross-talk, cancer progression, and opportunities for drug discovery [26,63]. For instance, using a complex model system, Gao et al., found that CAFs can form metastatic units with ascitic tumor cells and drive peritoneal metastasis formation, which is common in ovarian cancer [124]. Using such complex models can also identify mechanisms of chemo-resistance and pinpoint drug candidates more likely to be effective in vivo, since malignant cells often exhibit profound differences in sensitivity to therapeutic agents depending on the culture system utilized [125,126,127,128]. Furthermore, while patients receive well-defined cycles of chemotherapy, most studies, both in vitro and in vivo, do not mirror the concentrations and durations of treatment typical of clinical exposures.

In addition, preclinical examinations of chemotherapy-induced changes in the TME have been largely restricted to subcutaneous xenograft models that do not fully recapitulate all components of the TME. Studies using orthotopic or genetically engineered mouse models are needed to more fully understand disease processes. In HGSOC, most tumor cells harbor mutations in *TP53*, and mouse models have been recently developed with syngeneic murine ovarian cancer cells engineered to express mutant p53 protein via CRISPR/Cas9 [129]. Researchers must carefully consider candidate cell lines for their experiments, since unique and characteristic TMEs arise depending on the HGSOC model employed [130].

CAFs originate from numerous sources and are composed of discrete subpopulations. Therefore, it is difficult to generalize microenvironmental responses to chemotherapy due to heterogeneity within single tissues and between anatomic sites. For example, while cisplatin treatment may downregulate α-SMA in lung fibroblasts, studies in bladder and foreskin fibroblasts have found upregulation of α-SMA or no changes at all [24,35,44]. Furthermore, few studies have examined differences in CAFs between humans and other model organisms [131]. Research in the ovarian cancer TME has identified at least four different CAF subpopulations that express distinct molecular signatures characterized by variable expression of CD29, CD10, FAP, α-SMA, FSP1, PDGFR-β, podoplanin, and caveolin-1. This suggests that the use of single markers such as α-SMA to define CAF identity is imprecise. Embracing multiple CAF markers with complementary functional studies has the potential to elucidate whether therapy-induced changes reflect reprogramming of stromal fibroblasts or the selection of discrete populations. We anticipate that single-cell sequencing will serve to spatially and temporally resolve CAF subpopulations, as well as shed light on the epigenetic and evolutionary dynamics within specific microenvironments [132,133,134]. Advances in mass spectrometry-based proteomics have generated opportunities to characterize the tumor and stromal proteomes in situ [135], quantify the phosphorylation levels of critical signaling pathways [136,137], and identify secreted factors derived from both tumor and stromal cells [138]. In addition, imaging mass cytometry enables the multiplexed, spatial characterization of proteins in both tumor and stromal compartments and has already revealed important aspects of cellular communication and “cellular neighborhoods” in breast cancer [139].

Understanding how chemotherapeutics impact cell types in the TME other than CAFs may also inform opportunities for therapeutic intervention. Patients can experience varying degrees of myelosuppression, mucous membrane reactions, and alopecia, all evidence of unintended cellular targets [19,140]. In addition, taxanes and platinum-based agents can have significant neurotoxicity [141,142]. Because neurons interact with the TME in myriad ways, releasing neurotransmitters, peptides, and growth factors [143,144,145], there are likely to be unexamined roles for chemotherapeutic agents in the regulation of the communication between neurons and cancer cells [146]. Despite a growing literature showing that chemotherapeutics perturb nonmalignant TME cells, in most situations, the mechanistic basis of these effects remains unclear. As well as cross-linking DNA, cisplatin is also known to generate reactive oxygen species, lower GSH and NADH levels, disrupt calcium homeostasis, and activate ERK, JNK, and AKT signaling pathways [147]. PARP inhibitors may disrupt the roles of PARP in DNA damage repair and influence epigenetic remodeling. Several of these therapy-induced effects on fibroblasts are therefore unsurprising, whereas others require further elucidation. For example, the general upregulation of α-SMA may be due to cisplatin-induced changes in SMAD3 signaling, PAI-1 autocrine signaling, or other mechanisms [148]. To understand these aspects of treatment, future studies should investigate the transcriptional and translational changes as well as the epitranscriptomic and epigenetic remodeling that occurs in response to anticancer agents. How chemotherapeutic agents may remodel the pre-metastatic niche has not yet been explored.

Beyond their likely prognostic and diagnostic value [149], CAFs have garnered interest as targets of cancer therapy [150]. This could be of particular relevance to OvCa, since there are few targetable mutations. Rather than aiming to ablate whole CAF populations, it may be possible to reprogram CAFs towards an anti-tumorigenic phenotype [151]. Indeed, emerging research provides a rationale for targeting the TME with all-trans retinoic acid to induce CAF quiescence [152] or using CXCL12 receptor inhibitors to disrupt CAF-mediated immune evasion, as well as targeting many other CAF behaviors [153]. Fully realizing the clinical efficacy of these emerging chemotherapies will require a robust understanding of how common treatments influence the tumor microenvironment.

## 10. Conclusions

In summary, chemotherapy and radiation does not only affect the cancer cells, but has a profound imapct on the TME. Understanding the effect of various treatments on cancer cells is necessary to completely comprehend their action and develop novel therapeutics that interrupt undesired tumor–stroma interactions.

## Data Availability

Not applicable.
